# Influence of substrate thickness on cell-perceived stiffness: a computational study

**DOI:** 10.1007/s10237-026-02077-3

**Published:** 2026-06-03

**Authors:** Raphael Jakob, Alexander E. Ehret, Edoardo Mazza

**Affiliations:** 1 Institute for Mechanical Systems, ETH Zurich, CH-8092 Zürich, Switzerland; 2 Empa, Swiss Federal Laboratories for Materials Science and Technology, CH-8600 Dübendorf, Switzerland

**Keywords:** Hybrid discrete-continuum model, Cell-substrate depth sensing, Cell-perceived substrate stiffness, Cell-perceived substrate thickness

## Abstract

Cell culturing on soft substrates has become a widely applied method towards mimicking the compliant mechanical environment of cells *in vivo*, and substrate stiffness was shown to influence cell behavior. The cell-perceived stiffness, however, does not only depend on the modulus of the substrate material, but also on its thickness. In the present work, we propose an *in silico* approach to quantify the effect of substrate thickness on cell-perceived substrate stiffness. We coupled a discrete network representation of the cellular cytoskeleton including subcellular cell-substrate connections, i.e. focal adhesions (FA), with a continuum model of the substrate, and simulated the contraction of individual cells and cell patches. The results indicate that for substrates of heights larger than a multiple of the FA size (i.e. in the range of 5–10 μm) the cell-perceived displacements and forces depend only on the substrate material, i.e. the latter is perceived as a semi-infinite continuum. The FA displacements depend more strongly on substrate thickness than FA forces. Based on the present model with subcellular resolution, the length scale of FAs emerges as the governing length scale for depth sensing, which is independent of the dimensions of the cells or cell patches.

## Introduction

Cells interact with their environment through a range of mechanisms. They sense the biophysical properties of their extracellular matrix (ECM) through integrin-mediated adhesions (Kechagia et al. 2019), adapt their volume in response to physical confinement (Xie et al. 2024), and communicate with neighboring cells through paracrine signaling, force transmission and direct molecular exchange at cell–cell junctions (Lee et al. 2009; Hervé and Derangeon 2013). As an example of response to mechanical stimuli, cells detect stiffness gradients, migrating toward regions of higher rigidity in a process known as durotaxis (Espina et al. 2022).

*In vitro* experiments have become essential for fundamental studies in cell biology or for the identification of treatment targets, offering controlled platforms to analyze cell behavior. Yet, given the complexity of the *in vivo* microenvironment, researchers designing *in vitro* studies are confronted with a central question: Which aspects of the cell’s natural environment are most relevant to replicate as part of the *in vitro* culturing protocols? This may include control of fluid flow, pressure, osmolarity and, finally, substrate topography and stiffness. In the present study, we focus on the stiffness perceived by cells when cultured on elastomeric substrates.

The ECM stiffness in human organs spans from ∼1 kPa in brain tissue (Budday et al. 2015) to ∼10 GPa in cortical bone (Peralta et al. 2017; Zioupos and Currey 1998), and even if these reported values may be affected by the length-scale and technique of measurement (Wahlsten et al. 2023), the seven orders of magnitude impressively underline the range of mechanical environments cells can be exposed to.

As mentioned above, cells are highly mechanosensitive, probe their environment and use stiffness cues to regulate their behavior (Janmey et al. 2020). For instance, mesenchymal stem cells differentiate along distinct lineages depending on the rigidity of their substrate. Soft matrices (∼0.1–1 kPa) promote neurogenesis, intermediate stiffness (∼10 kPa) favors myogenesis, and stiffer environments ($\sim 30{-}40$ kPa) induce osteogenesis (Engler et al. 2006). Similarly, tumor cells exploit increased ECM stiffness in the tumor microenvironment to enhance invasion and metastasis (Kai et al. 2019). These observations underscore the critical role of matrix stiffness in directing cellular fate, properties and function, and underline the importance of choosing adequate mechanical surroundings for conducting *in vitro* experiments.

When investigating cell lines naturally residing in soft biological tissues, distinct differences can be observed when experiments are conducted on stiff substrates such as glass or tissue culture plastic compared to substrates with stiffness similar to *in vivo* settings (Discher et al. 2005; Achterberg et al. 2014). Therefore soft ‘biocompatible’ materials, such as silicone elastomers or hydrogels, are used with the aim of providing cell substrates whose stiffness is representative of the *in vivo* surrounding. While the modulus of such substrata can be determined by suitable mechanical testing (Norman et al. 2021; Reyes Lua et al. 2020), the stiffness perceived by individual cells in an *in vitro* experiment is also influenced by geometrical factors. Soft substrates are usually placed in glass or plastic containers of stiffness much higher than that of their content. Thus, the stiffness perceived by cells may be influenced by the rigidity of the stiff container when the compliant substrate is not thick enough. In the present work, we address the effect of substrate thickness on cell-perceived stiffness for single cells, cell clusters, and whole cell layers.

The influence of substrate thickness on cell response has been investigated with *in vitro* experiments. For example, Tusan et al. (2018) examined changes in cell area of osteogenic cells depending on substrate thickness and identified critical values of 3.2 μm for single cells and 54 μm for cell clusters cultured on a 1 kPa substrate, roughly indicating the length scales above which the effect of substrate height strongly reduces.

Mullen et al. (2015) found similar critical values of substrate thicknesses for the same cell type. Buxboim et al. (2010) investigated the cell response of mesenchymal stem cells cultured on gels with ∼1 kPa stiffness and determined moderate changes in depth specific cell spread area at 10-20 μm thickness and defined a characteristic tactile length of ∼5 μm. In contrast, Hernandez-Miranda et al. (2024) reported significant differences in cell spread area when culturing bone marrow stromal cells on substrates with 50 μm as compared to 200 μm thickness. The above studies used polyacrylamide hydrogels as substrates, a non-fibrous isotropic material. Material choice influences depth-sensing of cells. This was shown by Leong et al. (2010), who showed that mesenchymal stem cells behave significantly different on a collagen hydrogel of 130 μm thickness as compared to a gel with 1440 μm thickness. They attributed the increased depth sensitivity to the nonlinear strain stiffening properties of the collagen hydrogel that arises due to the fibrous network of collagen fibers.

In the present work, we perform a computational study to investigate the role of the height of an elastic substrate on both the forces and displacements occurring at FAs due to cell contraction. Several approaches were proposed to simulate the mechanical interaction between cells and the surrounding materials, e.g. (Schwarz and Soiné 2015a; Zündel et al. 2017, 2019; Wahlsten et al. 2023). The following computational studies specifically addressed the question of substrate depth sensing by cells: Leong et al. (2010) and Aghvami et al. (2016) found increased depth sensing capabilities of cells on fibrous substrates as compared to non-fibrous substrates by applying constant traction on a substrate region located at the cell periphery. Yang et al. (2023) modeled cells as a thin circular contractile disc perfectly adhered to an underlying substrate in a finite element context. They showed increased average interfacial substrate displacement with increasing substrate thickness, with significant changes for thicknesses smaller than the cell radius, i.e. 18 μm. Sen et al. (2009) created a continuum model including cytoskeletal components, a cortical shell, and the cell nucleus. Their results likewise indicate that depth sensing by cells is linked to substrate thickness values smaller than cell dimensions.

The main novelty of the present work lies in the fact that depth sensing is investigated based on a discrete model representation of single cells or cell clusters interacting at focal adhesions (FA) with an elastomeric substrate. This representation allows us to analyze the influence of substrate thickness at several length scales, from the size of isolated FAs, to the one of individual cells and cell clusters. This objective is pursued at the example of endothelial cells, which are cultured as individual cells, cell groups or a complete monolayer.

The present work thus overcomes limitations of previous models characterizing cell-substrate interaction on a cell scale based on an assumption of perfect adhesion throughout the cell-substrate interface. FAs are protein complexes of few microns in length located primarily at the periphery of adherent cells (Kim and Wirtz 2013; Chen et al. 2003). They bind to the substrate underneath the cell on one end and to the contractile actin cytoskeleton on the other one. Cell-substrate interaction therefore takes place in a highly localized manner. Solowiej-Wedderburn and Dunlop (2022) recently proved the influence of interaction localization by implementing a model of a single contractile cell while modifying the contact interface between cell and substrate from a disc to a ring and discrete spots. They found that for different adhesion patterns, cells perceive different substrate stiffnesses. We hypothesized that localized interactions influence to an even larger extent the deformation behavior of cell clusters or whole cell layers. Our new computational approach allows us to quantitatively investigate the length scale which governs substrate depth sensing by the contracting cell, for single cells and cell patches.

## Methods

### Discrete network representation of the cell

The intracellular cytoskeleton is a complex network of protein fibers composed of three parts: the actin cytoskeleton, the intermediate filaments, and the microtubules (Fletcher and Mullins 2010). Among these, the actin cytoskeleton plays a central role in cell’s contractile properties (Vicente-Manzanares et al. 2009). Phosphorylation of non-muscle myosin II, which forms actomyosin complexes with actin, leads to contraction of actin networks (Chinthalapudi and Heissler 2024). The actin cytoskeleton is thus the most important contributor to cell-substrate mechanical interaction.

**Fig. 1:**
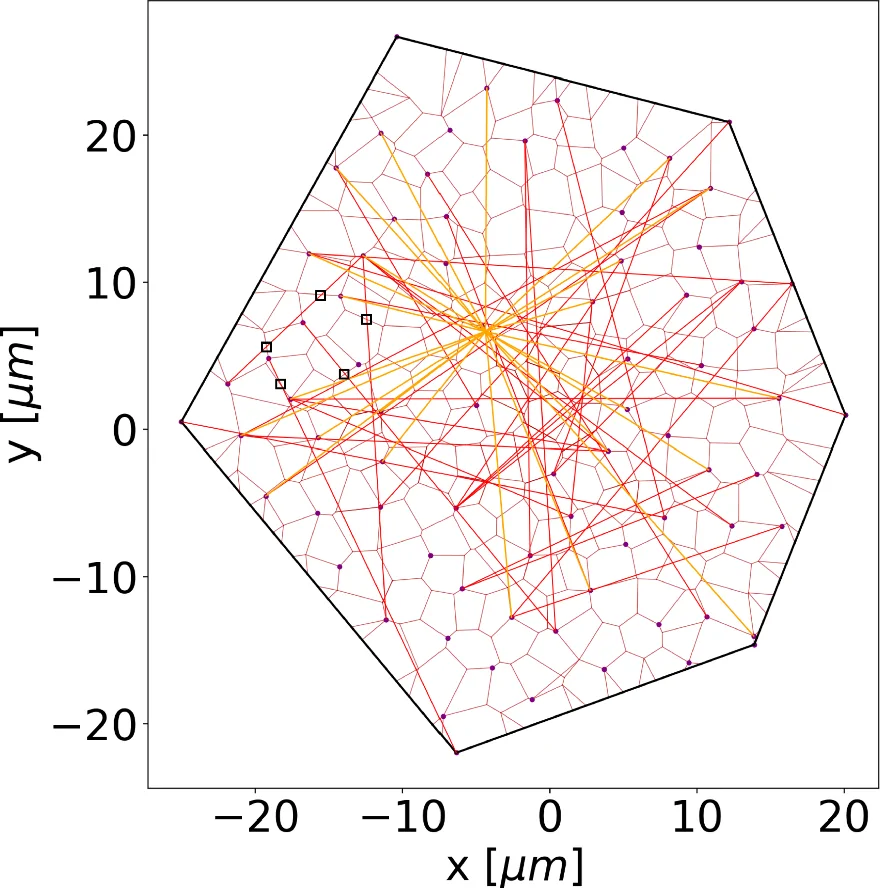
DNM of a single cell with representations of cell membrane (black), cortical actin (dark red), ventral stress fibers (red), perinuclear stress fibers (orange) and FA (purple dots). Selected links between ventral and cortical fibers are included as black squares for illustration purposes

**Fig. 2:**
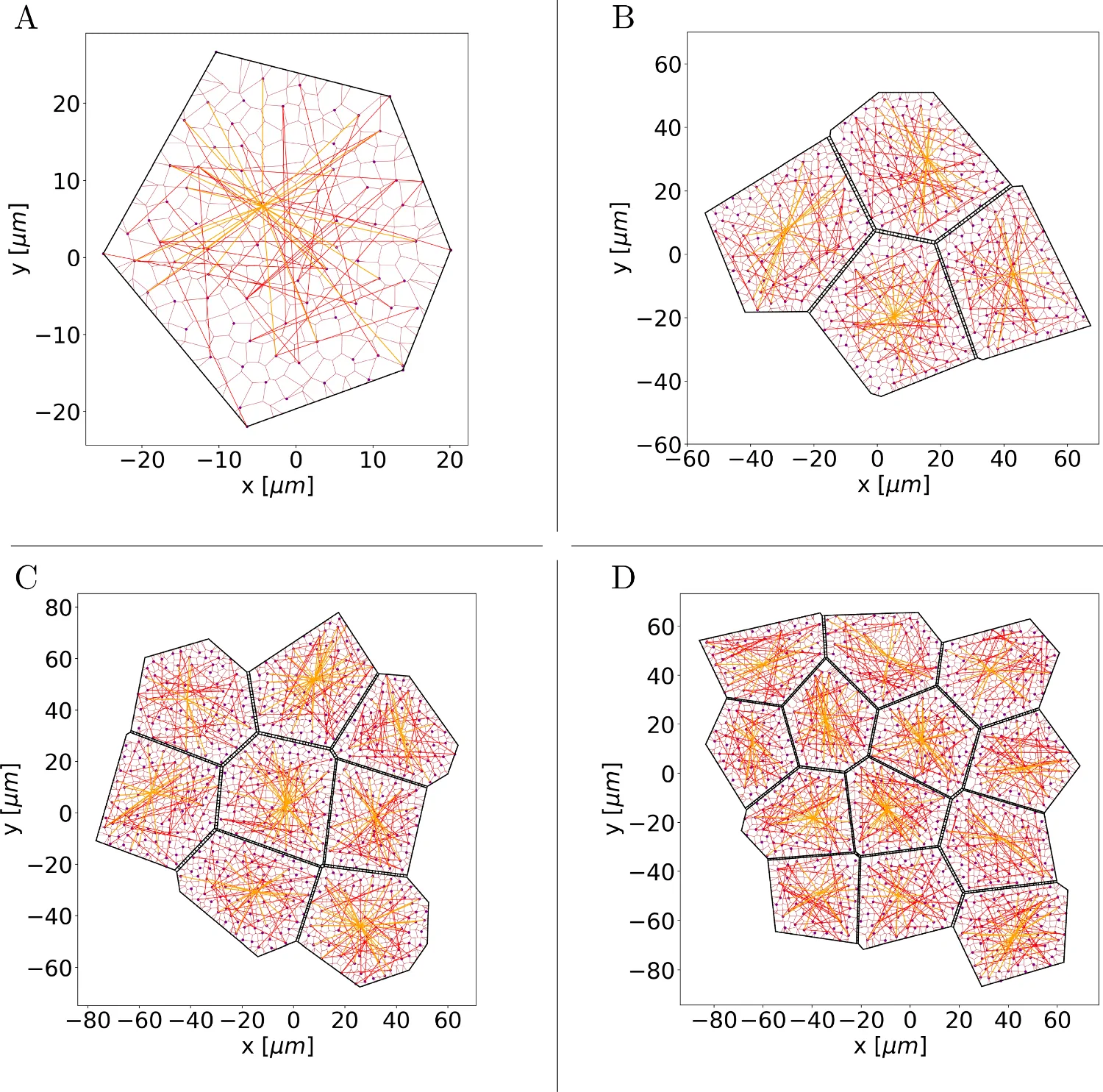
DNMs of simulated cell models: single cell (**A**), cell patch of 4 (**B**), 8 (**C**), and 13 (**D**) cells. Fiber representations are cell membrane (black), cortical actin (dark red), ventral stress fibers (red), perinuclear stress fibers (orange) cell-cell junctions (black), and FA (purple dots)

The cell model used in this study is based on our previous work, Jakob et al. (2024); Choi et al. (2024); Jakob et al. (2025). It contains representations of the cellular and nuclear membrane, ventral and perinuclear stress fibers, the actin cortex, FAs, and cell-cell junctions. The cellular membrane is defined by network connections lying on the edge of a convex polygon. The actin cortex is represented with a Voronoi mesh and is connected to the cellular membrane.  The seed points for the creation of the Voronoi mesh were created by placement of an evenly spaced point grid within the cellular domain with an interdistance of 2.5 μm and subsequent random perturbation of these points in horizontal and vertical direction of up to 1  *μ*m. Ventral stress fibers are included as long connections spanning the cellular floor linking two FA sites with each other. They connect also to the cortical actin cytoskeleton (Vignaud et al. 2021), and this coupling is modeled by randomly selecting 1 to 10 points where the stress fiber and cortical actin intersect and inserting a node at that point for each ventral stress fiber. For the creation of perinuclear stress fibers, a hypothetical nucleus was included as an ellipse within the cell. Its center was then selected as a pivot point lying on top of the nucleus and the pivot was then connected with FA outside of the ellipse. FA were defined by selecting a random subset of network nodes, with density consistent with values in literature (Nair et al. 2021). The thus resulting discrete network model (DNM) of a single cell and its dimensions are shown in Fig. 1. To generate clusters of cells, many of these DNM cell representations were placed next to each other and connected with a cell-cell junction connection, similar as for the coupling of fibers and substrate at FAs (Jakob et al. 2024). Examples of the thus resulting cell patches are illustrated in Fig. 2.

All fibers were represented as bilinear springs with the following energy potential (see Jakob et al. 2024)

1 $$\begin{aligned} E = \frac{1}{2}\left[ K_{\mathrm t} \bigg \langle \frac{r-r_{\mathrm a}}{r_{\mathrm a}} \bigg \rangle ^2 + K_{\mathrm c} \bigg \langle \frac{r_{\mathrm a}-r}{r_{\mathrm a}} \bigg \rangle ^2\right] . \end{aligned}$$

Here, $K_\textrm{t}$ and $K_\textrm{c}$ stand for tensile and compressive fiber stiffness, respectively, *r* stands for the current fiber length, $r_a$ is the zero force length of the fiber, and *E* is the stretch-dependent length-specific energy density with respect to the fiber’s zero force length. The symbols < and > represent Macaulay brackets. To model the contractile behavior of the actin cytoskeleton due to non-muscle myosin II, a multiplicative split of the fiber stretch (cf. e.g. Martins et al. 2006) was utilized as

2 $$\begin{aligned} {r} = \lambda _{e}\, a \,r_0 = \lambda _{e} \,r_{a}. \end{aligned}$$

Here, $r_0$ is the fiber length as created in the DNM and a<1 is the activation parameter, leading to fiber tension in the initial state with subsequent cellular contraction. The values of the respective parameters (Table 1 ) were selected based on experimental observations on endothelial cells, including traction force microscopy data (Jakob et al. 2024). The thus resulting network connections only transmit forces in axial direction, i.e. its network nodes represent freely rotating joints.

The DNM described above results in a sub-isostatic network (Picu and Ganghoffer 2020) with coordination number, i.e. an average number of bonds per node, only slightly above 3. To mechanically stabilize the network, additional ‘auxiliary’ connectors were introduced until all network nodes had a connectivity of at least 5 (see Mauri et al. 2016). Note that in the 3D case, isostatic networks require a network connectivity of at least 6. However, in the present case, while the DNM is implemented in a 3D model, the network is created in a plane parallel to the substrate surface. Thus, a connectivity of 5 was sufficient to achieve solver convergence. Network connections were represented in Abaqus/CAE (Abaqus 2023, Dassault Systèmes) as one dimensional connector elements (CONN3D2) with elastic characteristics according to Table 1 and a very small viscous contribution characterized by a linear dashpot. These stabilizing connectors had zero elastic stiffness and the same small viscous contribution and were required to enable a robust solution of the problem by means of the finite element solver. This allowed us to preserve the ability of the model to well represent experimental values, as the model parameters had been validated in Jakob et al. (2024) based on an implementation that did not require the addition of such connectors. A parameter study showed negligible effect of the viscous part on the model solution.

As explained in Jakob et al. (2024), the fiber stiffness and activation level of ventral and perinuclear stress fibers was defined based on the values reported in Deguchi et al. (2005). For the cortical actin filaments the stiffness was selected based on the data reported in Kojima et al. 1994 while the activation was defined according to Vignaud et al. (2021). Based on their slenderness, the compressive stiffness of all fibers was assumed to be only 10 % of the respective tensile value. The membrane’s stiffness contribution was assumed to be negligible and included mainly for tracing cell boundaries. The number of fibers within each cell was set based on comparisons with area strain values from traction force microscopy experiments performed with individual endothelial cells in Lúa (2020).

While the cell model is 2D, i.e. planar, out-of-plane force components were considered for the perinuclear stress fibers, as explained in Sect. 2.2.

**Table 1:** Tensile (t) and compressive (c) stiffnesses of fibers in the DNM as well as their activation parameter. The membrane was modeled as non-contractile and therefore $r_0=r_a$ for the membrane

	$K_{\mathrm t}$ [nN]	$K_{\mathrm c}$ [nN]	*a* [−]
Actin cortex	29.1	2.91	0.96
Ventral & perinuclear stress fibers	20.2	2.02	0.848
Cellular membrane	0.1	0.01	–

### Finite element model of the substrate and cell-substrate interaction

**Fig. 3:**
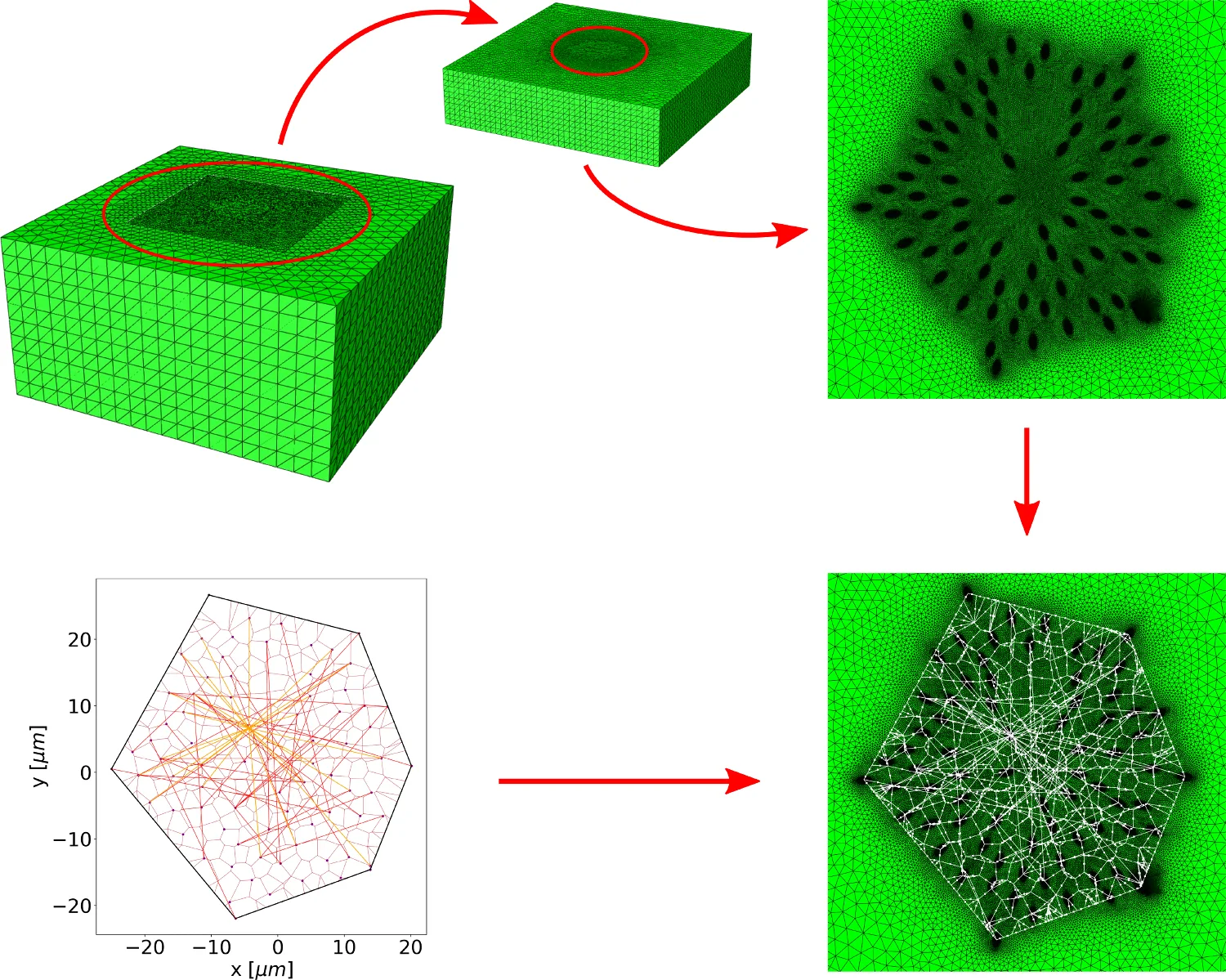
Coupled DNM-substrate model. The whole substrate (upper left), here with thickness of 200 μm, is split to allow for a refined mesh in an inner region (upper middle). Ellipses are then placed in the center of that inner region (upper right) at the location of FA network nodes gathered from the DNM (lower left). The DNM is then imported into the substrate model and placed on top of the substrate surface (lower right)

The flexible substrate was represented as a cuboid with different thicknesses in Abaqus. Its lateral dimensions were 200×200 μm, while its height was varied from 5  to 200 μm. The case with 0 thickness was considered using a substrate with high stiffness. Separate meshing was defined for a central region at the cuboid’s surface of 100×100 μm and thickness varying from 2  to 50 μm. It was tied to the cuboid’s outer part via kinematic tie constraints. An example of such a substrate model is shown in Fig. 3.

The outer surfaces, except the top one, were fixed using zero displacement constraints in all directions. To assess the effect of using these constraints for the sides compared to larger substrate dimensions, an additional simulation was conducted with the opposite limit case of fixed bottom but free side surfaces (data not shown). The obtained relative change in the relevant results below 0.1 % suggested that the chosen boundary conditions and substrate dimensions were appropriate for the studies performed here.

Cells adhered to the substrate were modeled by placing a cell or cell patch DNM in the central region of the substrate surface. The connection of the DNM to the substrate was realized at FAs represented as ellipses with 2 μm major and 1 μm minor axes at the corresponding substrate locations (cf. Zündel et al. 2017), specified through the DNM (purple dots in Fig. 1). The substrate nodes within the ellipses were coupled to the FA nodes of the DNM though the ‘distributing coupling’ option of the FE software. The orientations of the ellipses were chosen in such a way that their major axis pointed toward the center of the cellular domain as defined by the DNM. To avoid overlap of ellipses and subsequent issues with meshing, the minimal interspace distance of FA nodes in the DNM was set to 3 μm. The cuboid was meshed with linear tetrahedral elements (C3D4). The seed points were set so that an element size of ∼0.15 μm was achieved within and around the ellipses. A mesh refinement study comparing simulations of a single cell with a refined mesh (element size of ∼0.1 μm within ellipses) led to changes in mean FA displacements of less than 3 % and in the mean force at FAs of less than 2 % for all substrate thicknesses investigated. Thus, the model was considered to be sufficiently discretized. A neo-Hookean hyperelastic material model was selected to represent the substrate’s mechanical behavior. The parameters were chosen to yield a Young’s modulus of 7 kPa and a Poisson’s ratio of 0.45, in line with the properties of soft elastomers typically used as substrate materials Lúa 2020; Jacot et al. 2008; Schwarz and Soiné 2015b.

The FA points of the DNM situated in the middle of the ellipses on the substrate surfaces were connected to the substrate by means of a distributing coupling between FA point and nodes within the ellipse surface (Fig. 3).

The pivot point connecting all perinuclear stress fibers on top of the nucleus was elevated from the substrate surface by 5 μm. This value is in agreement with typical maximal cell heights at the nucleus of endothelial cells (Yamada and Mouri 2010; Birukova et al. 2009; Sato et al. 2000). The elevated position of the pivot point was selected to enable the representation of out of plane force interactions between cell and substrate. In cells, perinuclear stress fibers bind to the cellular floor, while enveloping the nucleus (Maninova et al. 2017). Thus, when contracting, the fibers pull on focal adhesions and push the nucleus onto the substrate. To model the out of plane force transmission, the pivot point was connected with a set of substrate nodes located below the nucleus. Nodes were part of the subset if they were located within an ellipse with minor and major axes of random length between 4 and 7 μm and random orientation underneath the nucleus, Activation of perinuclear stress fibers thus caused tensile forces at focal adhesion with a vertical component and corresponding downward forces were transmitted to the substrate at the nodes within the ellipse underneath the nucleus.

### Data analysis from simulations of cell contraction

Activation of fiber contraction leads to reduction of the projected area of cells on the substrate. To quantify area reduction, we tracked the displacement of the substrate surface nodes, which in the initial state are underneath the cell. A concave hull was placed around all these substrate surface nodes, using scipy’s alphashape method (Virtanen et al. 2020). The area was then evaluated with python’s shapely package. Cell area changes upon contraction were quantified for a single cell and cell patches of 4, 8, and 13 cells on substrates with thicknesses of 200, 100, 50, 10, and 5 μm, as well as on a substrate with very high stiffness (MPa range) to simulate zero substrate thickness, i.e. culturing on glass or tissue culture plastic.

To quantify local values of area reduction in cell patches, the area strain was calculated using initial and final nodal coordinates to calculate the surface area of the triangular elements on the substrate surface. Area strain of each surface element was calculated as

3 $$\begin{aligned} \varepsilon _A = \frac{A_{\textrm{final}}}{A_{\textrm{init}}}-1. \end{aligned}$$

Here, $A_{\textrm{init}}$ and $A_{\textrm{final}}$ denote the initial and final area of the surface triangle of each surface element, respectively. Negative $\varepsilon _A$ values indicate area contraction while positive values indicate area increase.

The forces resulting from the contractile cytoskeletal network are transferred to the substrate at FAs. These interface forces were quantified as the forces resulting at FA nodes on the DNM side, i.e. the resultant forces of all connections with endpoints at specific FA nodes. Mean values of forces at all FAs within single cells or cell patches were quantified.

### Models of single FA and contracting discs

To study the effect of a discrete vs. continuous representation (cf. Yang et al. 2023), we also developed finite element models of a single FA as well as contracting circular discs, representing a cell or cell patches. In all simulations, the substrate material properties were defined through the same hyperelastic model as in Sec. 2.2.

**Fig. 4:**
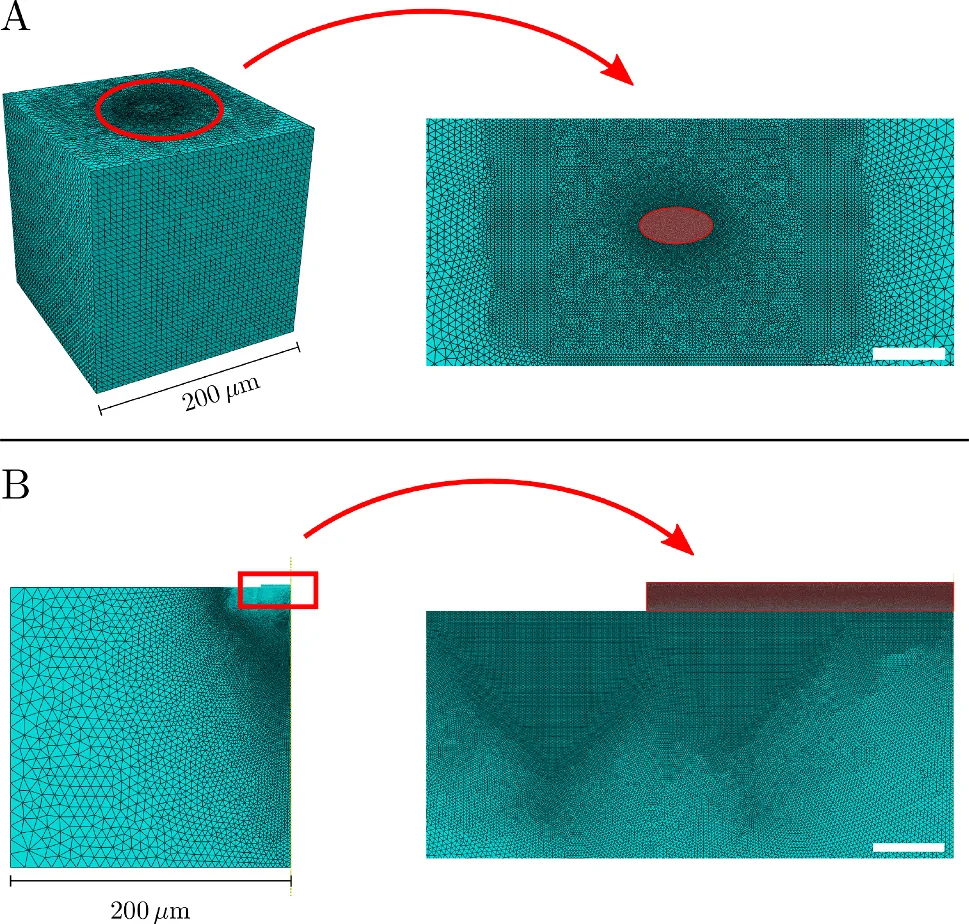
Modeling of a single FA (**A**) and a contracting disc (**B**) on an elastomer substrate. **A** FA simulated on a cuboid with 200 μm side length. The elliptical FA is highlighted in red in the magnified view on the right. Scale bar: 2 μm. **B** Axisymmetric contracting disc model. The substrate was represented as a cylinder with 200 μm radius and 200 μm height. The disc is highlighted in red in the magnified view on the right and has the same area as the single cell DNM. Scale bar: 5 μm

The single FA was represented similarly as in the DNM simulations as an ellipse with 2 μm major and 1 μm minor axis on a cuboid substrate with a square surface area of 200×200 μm and variable thickness. All outer surfaces of the cuboid except the top surface were fixed (encastre). The ellipse was coupled via the ‘distributing coupling’ option to a reference point located in the center of the ellipse, on which a force was imposed. The mesh discretization was chosen such that an average element size of $\sim 0.1\,\mu$m was obtained in the elliptical area of the FA. The resulting FA displacement was characterized by the displacement of the reference point. Figure 4 shows the model for a substrate thickness of 200 μm.

The model of a contracting circular disc was realized using an axisymmetric representation. The substrate was thus modeled as a cylinder with a radius of 200 μm and variable thickness. The disc representing a single cell or cell patches was modeled as a cylinder with a constant (arbitrary) thickness of 2 μm and a radius of 21 μm representing a single cell with the same area as the single cell DNM (Fig. 2A), and 46, 65, and 74 μm representing cell patches with the same patch area as the DNM of the cell patch with 4, 8, and 13 cells, respectively (Fig. 2B–D). The resulting model for a disc representative of the single cell is shown in Fig. 4B. The cylinder was tied to the substrate at their interface using surface to surface constraints. Both parts were meshed with triangular CAX3 elements. To assess the mesh quality, the element size at the interface on both the disc and substrate side was changed by a factor of two. This led to differences in results of less than 1 %.

Preliminary simulations indicated that in order to obtain similar contraction behavior, a linear elastic material with elastic modulus of 14 kPa and Poisson’s ratio of 0.45 could be chosen for the disc. Cell contraction was implemented by exploiting the thermo-mechanical coupling implemented in the software. To this end, the isotropic expansion coefficient α of the disc material was fitted such that with a unit temperature decrease the resulting area reduction on a substrate with 200 μm thickness was equal to the corresponding area reduction with the hybrid model. This procedure resulted in values for α of 0.041 K$^{-1}$ for the single cell and 0.031 K$^{-1}$, 0.032 K$^{-1}$, 0.033 K$^{-1}$ for the cell patches with 4,8, and 13 cells, respectively.

Fixed (encastre) boundary conditions were applied to the bottom and the mantle of the substrate cylinder. Cell contraction was modeled by linearly decreasing the temperature by 1 K over the simulation time. For both, single FA and disc, simulations were performed with substrates of varying thickness, as for the coupled model

## Results

### Cell area contraction

**Fig. 5:**
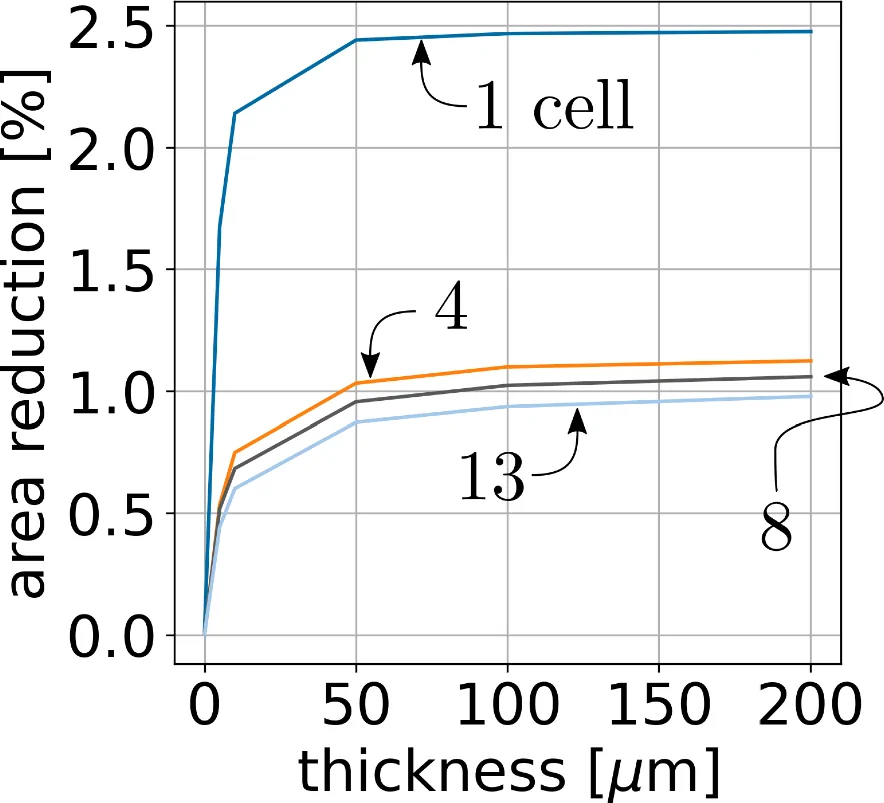
Area change of a single cell and cell patches of 4 , 8 and 13 cells

To investigate the cell response to a change in substrate thickness in experiments, the change of cell area is a useful measure obtained from immunofluorescence images. In the literature, several fold differences in cell area depending on substrate thickness are reported (Tusan et al. 2018; Buxboim et al. 2010; Mullen et al. 2015). To quantify the dependence of area contraction on substrate thickness, simulations of contracting cells and cell clusters on flexible elastomer substrates with varying thickness were conducted in this study. The results are reported in Fig. 5.

Cell area reductions are negligible for rigid substrates (zero thickness) while they increase for thicker elastomers. However, the values remain below 2.5 % for all cells and cell patches investigated here, thus orders of magnitude smaller than cell area differences reported in literature for different cell-scale stiffnesses (Tusan et al. 2018; Buxboim et al. 2010; Mullen et al. 2015).

Area reduction is largest for the single cell case. All configurations display a similar sensitivity to substrate thickness, which is analyzed considering forces and displacements at FAs in the next section.

### FA forces and displacements in single cells and cell patches

**Fig. 6:**
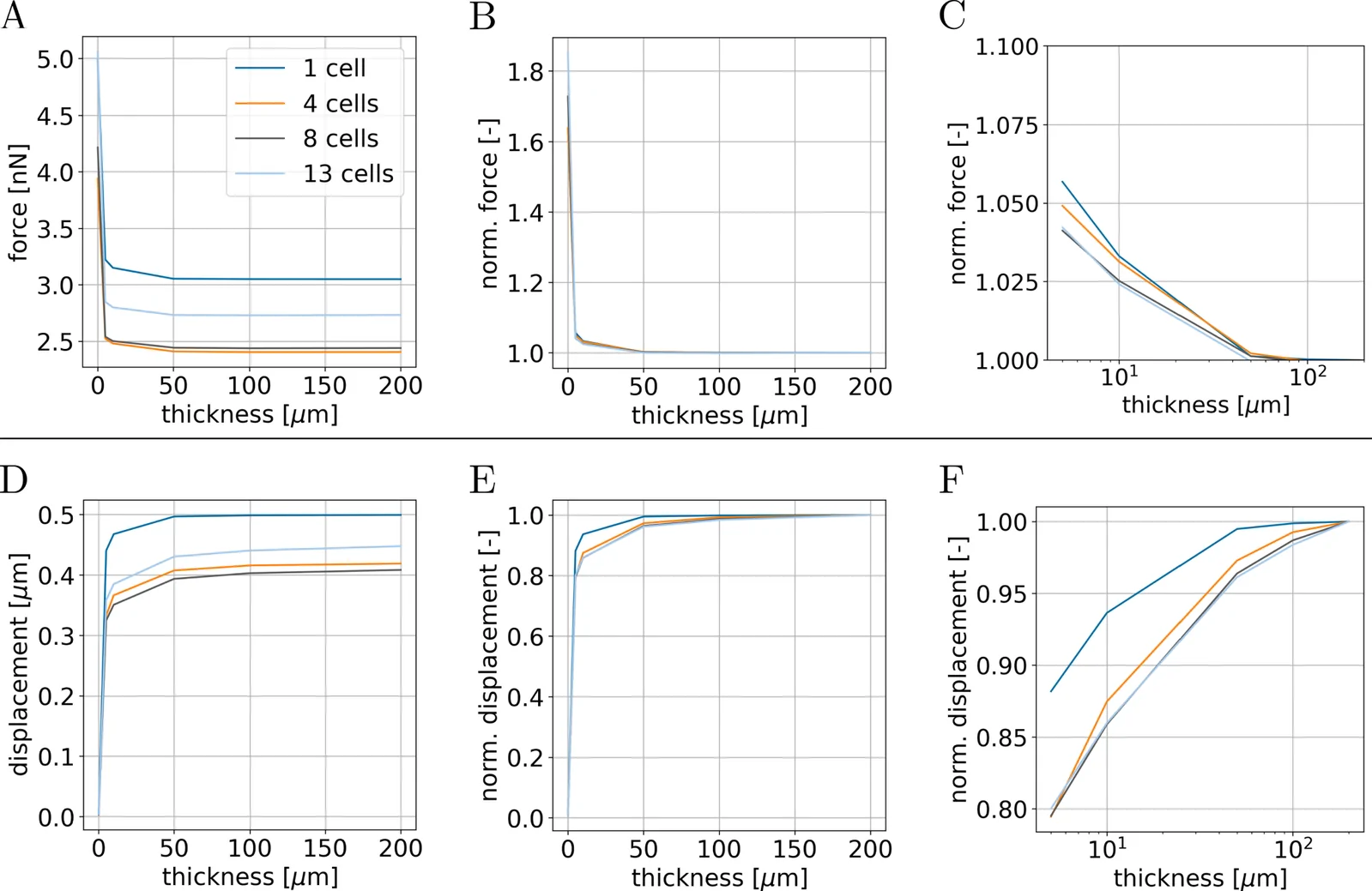
Mean values of forces (**A**, **B**, **C**) and displacements (**D**, **E**, **F**) at FAs in cells and cell patches with 4 cells, 8 cells, and 13 cells. Reported are the mean absolute values (**A**, **D**), the values normalized to the behavior on a substrate with 200 μm thickness (**B**, **E**), and the same data in a semi-logarithmic representation (**C**, **F**)

The force and displacement characteristics of FAs in single cells and cell patches upon contraction are reported in Fig. 6 as mean values for each case considered.

The forces in FAs calculated for rigid (zero thickness) substrates increased on average by 70 % compared to those on a substrate with 200 μm thickness. This effect rapidly decreases with substrate thickness, and convergence to the mean forces and displacements at 200 μm is observed, so that the latter is indeed representative of a substrate with infinite thickness in the present case (Fig. 6A,D).

In order to analyze the factors by which forces and displacements at FAs are altered compared to this limit case, these quantities were normalized by their respective values on a 200 μm substrate (Fig. 6B,E).

The normalized FA force represented against substrate height in a logarithmic scale reveals that it is only weakly sensitive to the substrate thickness, with maximal changes of about 5 % between the substrates of 5 μm and 200 μm height. This result is nearly independent of the patch size, with merely slightly higher values for single cells and the smallest cell patch observed here.

**Fig. 7:**
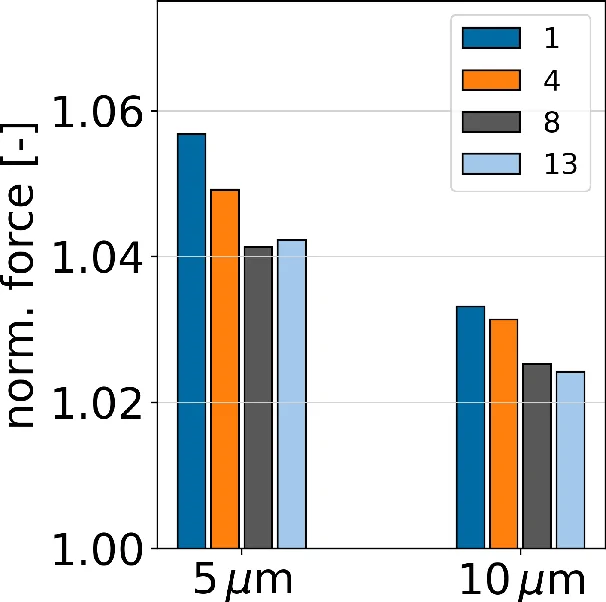
Normalized FA forces for single cell (1) and increasing cell patch sizes (4,8,13) on substrates with 5 and 10 μm thickness

Figure 7 shows a more refined representation of the normalized force for substrates of 5 and 10  μm thickness, and highlights that the corresponding reductions in height by factors of 40 and 20 lead to increases in mean FA force in the range of 5 % and 3 %, respectively, both for single cells and all patches.

The differences between single cells and patches are more pronounced for the FA displacements (Fig. 6D,E,F). Particularly the normalized values (Fig. 6E,F) reveal clear differences between individual cells (dark blue line) on the one hand and cell patches on the other one, whose response rapidly converges to a consistent curve with increasing patch size. For the single cell, notable differences characterized by a change of 10 % of their magnitude are obtained for substrates thinner than about 5 μm. For the patches, independent of their sizes, FA displacements are reduced by 10 % already for thicker substrates in the order of 10–20 μm.

**Fig. 8:**
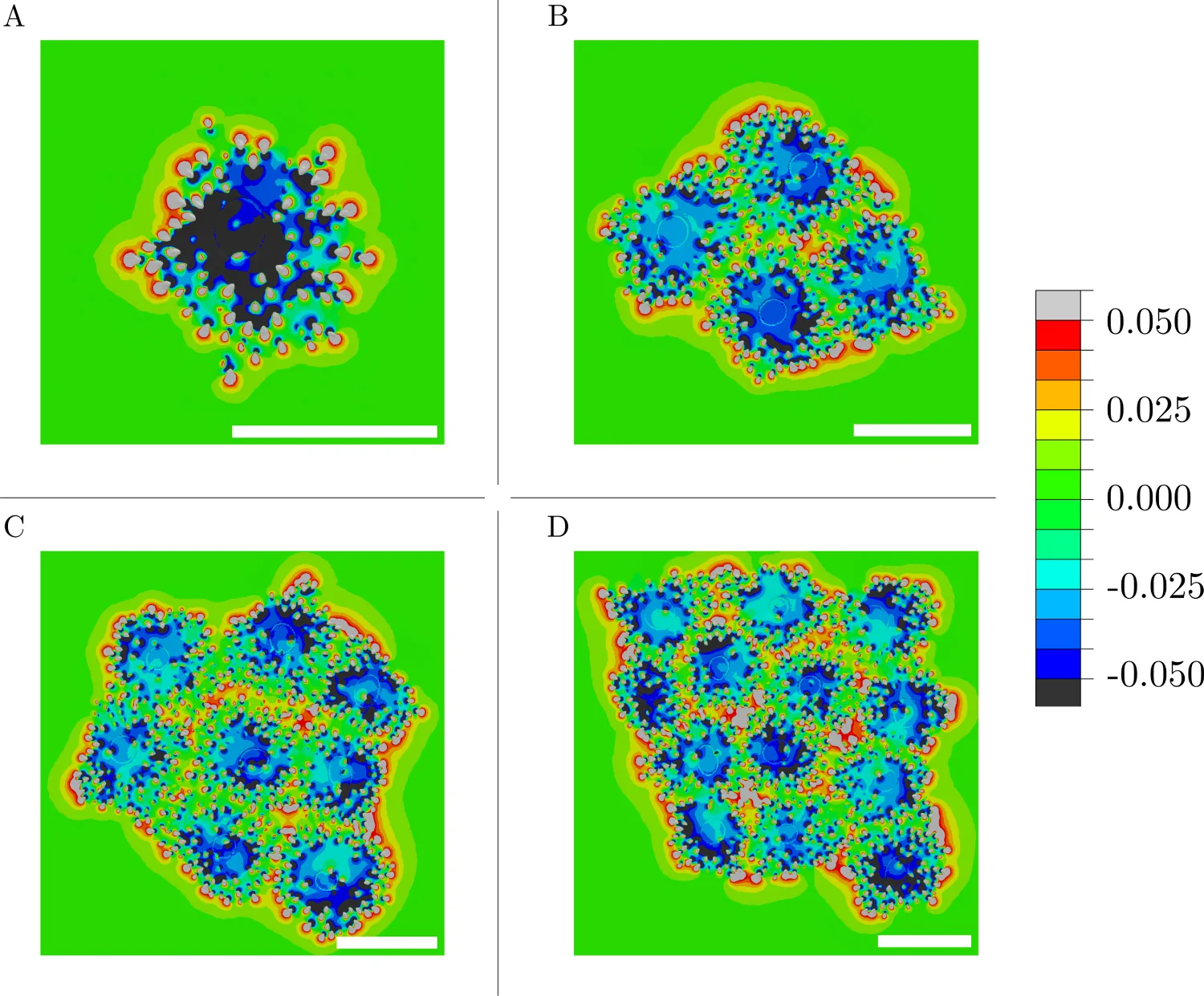
Area strain of single cell (**A**) and cell clusters with 4 cells (**B**), 8 cells (**C**), and 13 cells (**D**). Scale bar: 40 μm

To better understand the dependence of FA displacements and forces on cell patch size, we analyzed the strain field in the substrate underneath the cell patches. Figure 8 shows the area strain as defined in Sect. 2.3 for the single cell and cell patches. For a single cell (Fig. 8A) pronounced heterogeneity is visible, with highest and lowest magnitudes at FA sites depending on the radial coordinate. This is in line with the expectation that in a contracting cell FAs displace concentrically, thus leading to negative area strains radially just inside of the adhesion site, and positive area strains just outside. Even though heterogeneity is visible, generally negative area strains are observed underneath the cell away from FA sites, and positive area strains just outside of the cellular domain. This deformation profile will thus be termed “coordinated concentric contraction". Interestingly, the deformation induced by cell patches is not a scale-up of the pattern observed for a single cell (Fig. 8B–D). Rather, it represents a juxtaposition of the pattern of individual cells. The deformation is highly heterogeneous, exhibiting contractile states underneath cells and tensional states in intercellular domains. Thus, the global deformation of cell patches is fundamentally different from that of individual cells and may be termed “competitive distributed contraction", as adjacent contracting cells provide mutual resistance to contraction resulting in the tensile regions between cells.

**Fig. 9:**
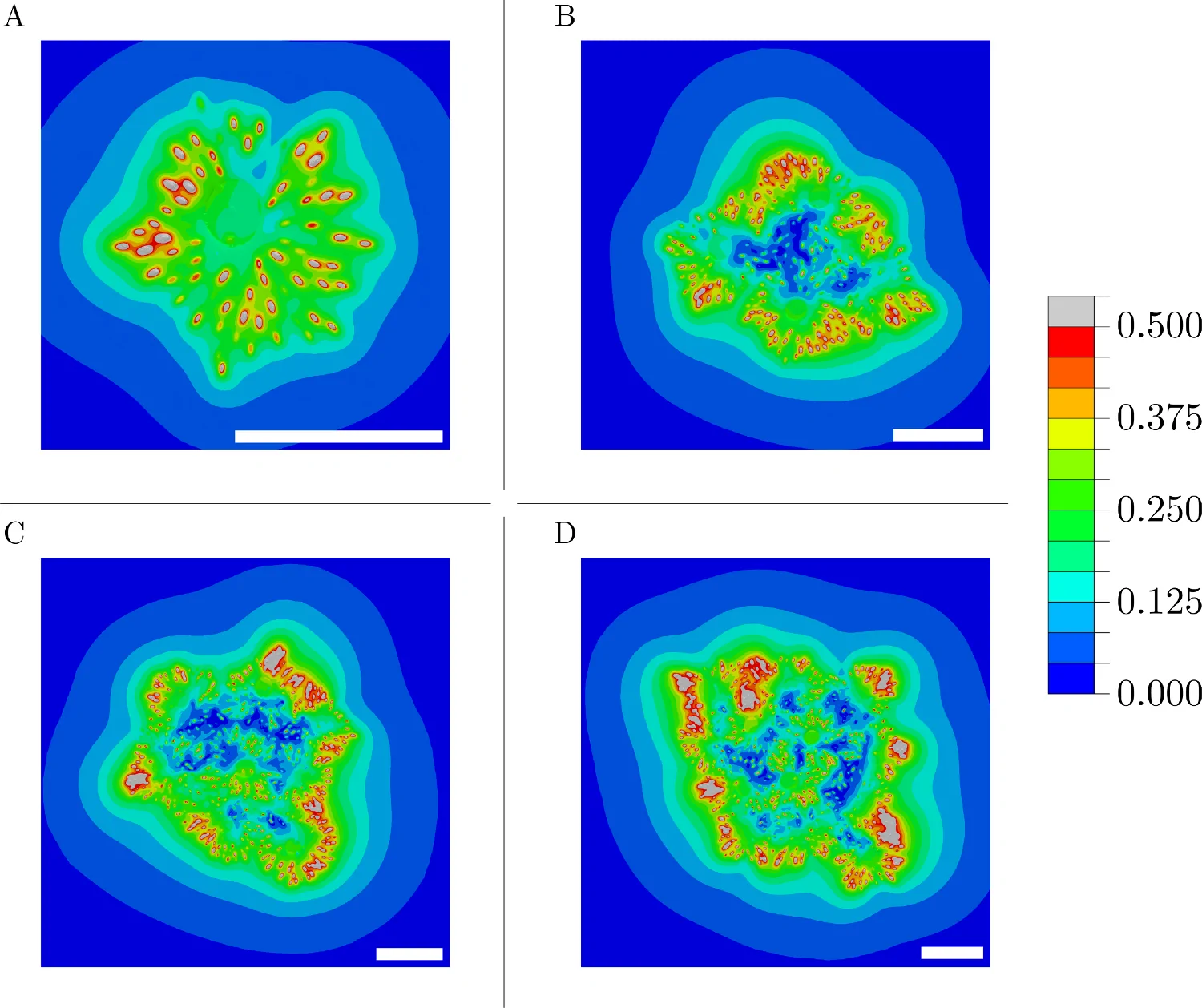
Displacement magnitudes on the substrate surface induced by the contracting cell (**A**) and cell patches of 4 cells (**B**), 8 cells (**C**) and 13 cells (**D**). Scale bar: 40 μm. In each image the emergence of clusters of FA with similar displacement magnitude can be recognized, with a mean cluster size in the range of few tens of micrometers

The difference in contraction behavior between single cells and cell patches is also visible when considering the magnitudes of the contraction-induced displacements on the substrate surface (Fig. 9). The heterogeneous contractile and tensional states in the cell patches lead to areas in the inner patch domain that experience almost no displacement. The largest displacements for the cell patches are experienced in the outermost parts of the patches, forming a ring of high substrate displacements around an inner domain with much smaller values.

These results indicate that depth sensitivity cannot scale with the dimensions of the contractile elements, from individual cells, to cell patches, to cell layers. Thus, the model representation of distributed FA forces as a contracting disc of varying size is expected to reveal a different sensitivity of FA forces on substrate height than the coupled DNM-substrate models studied here.

### Thickness sensitivity of a single FA and contracting discs

**Fig. 10:**
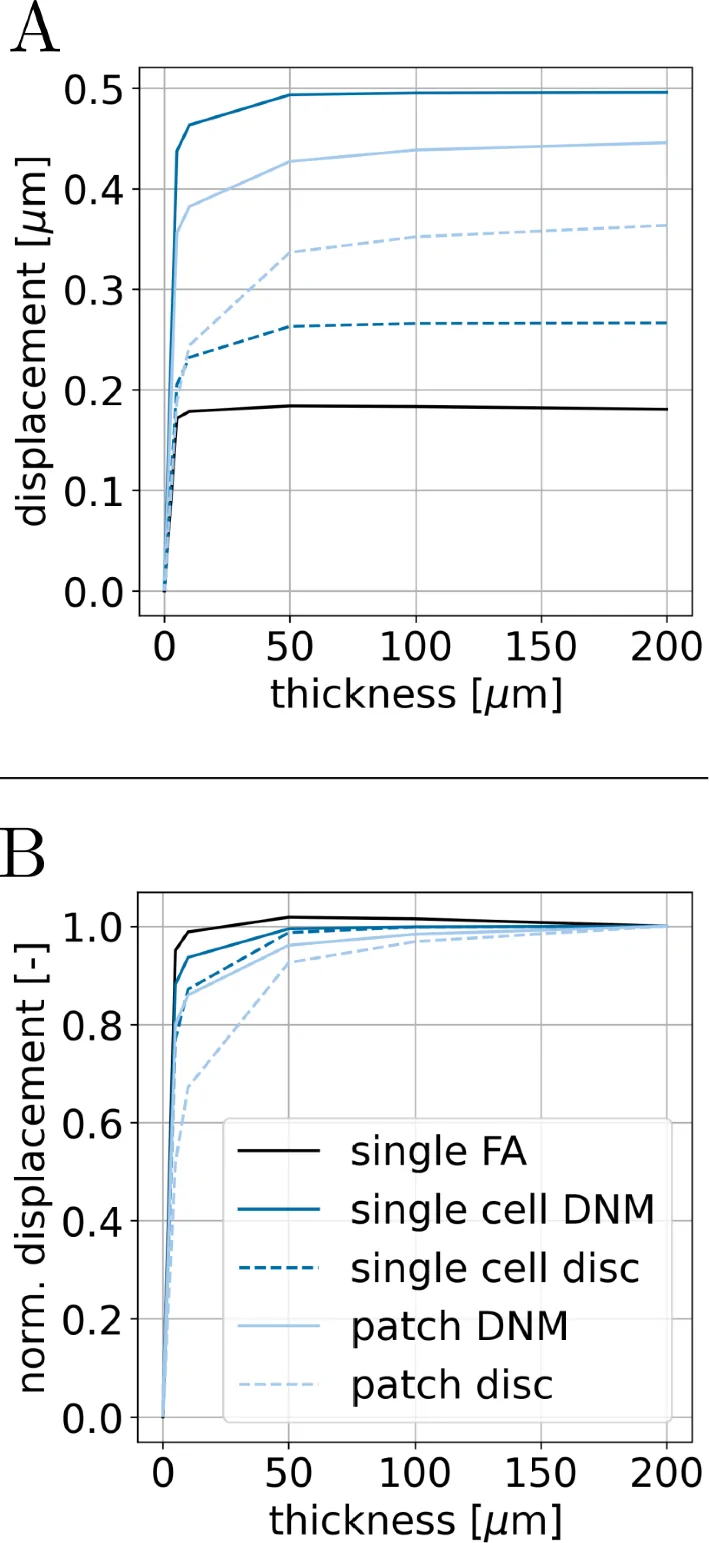
Displacement behavior of single FAs, FAs in DNMs, and contracting disc models of single cells and cell patches, in terms of absolute values (**A**) and values normalized to the displacements of a substrate with thickness of 200 μm (**B**). The single FA (black) experiences the force equal to the mean force of the FA in the single cell model as given in Fig. 6A

The results obtained from the contracting coupled models were compared to the deformation behavior of a single FA on the one side, and the one of discs representing a continuum model of contracting cells on the other. Results are reported in Fig. 10.

**Fig. 11:**
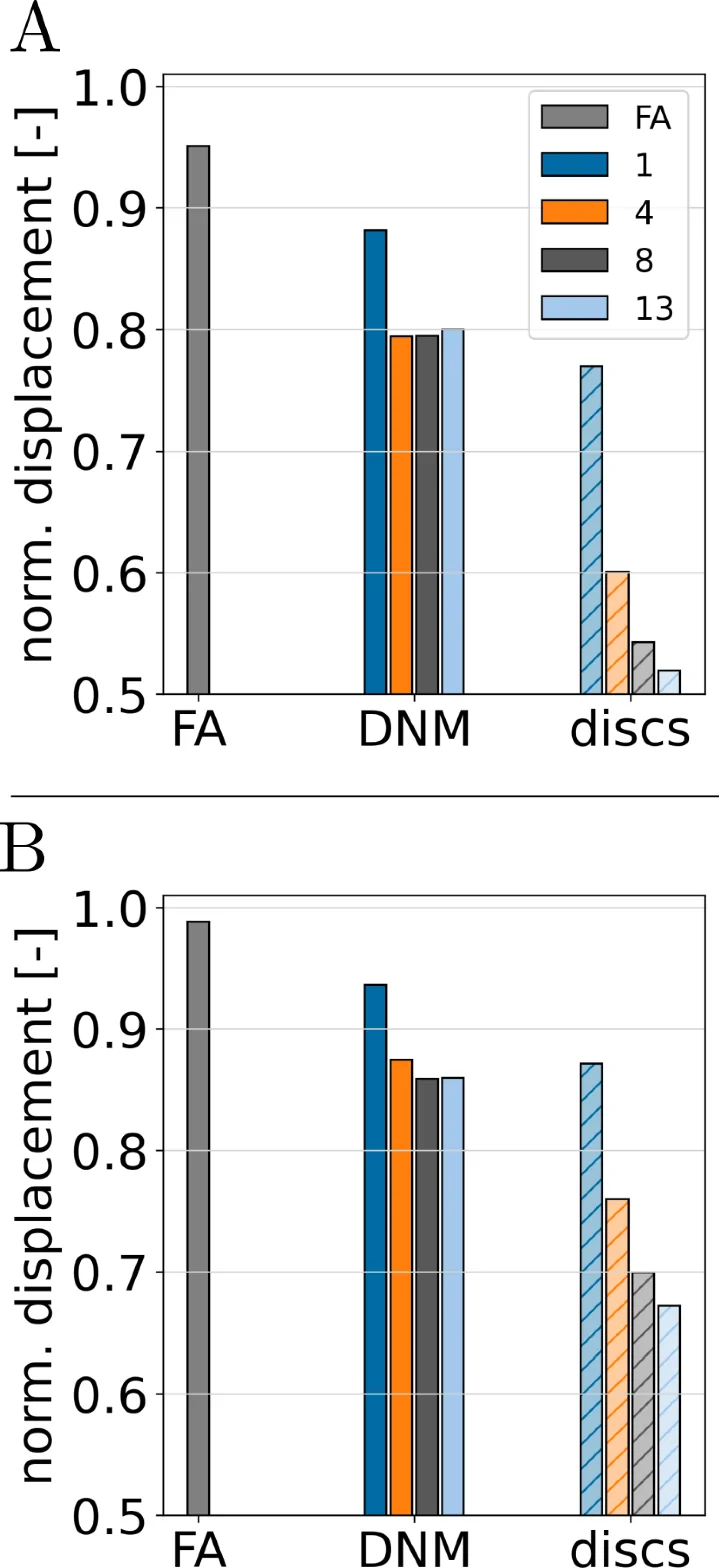
Normalized displacements of an isolated FA, FAs in coupled models of 1, 4, 8, and 13 cells (left to right), and equivalent contracting discs for 1, 4, 8, and 13 cells (left to right) on a substrate with 5 μm thickness (**A**) and 10 μm thickness (**B**). The equivalent disc models exhibited the same area and area change due to contraction on a substrate with 200 μm thickness as their DNM counterparts

Due to their stronger sensitivity on substrate height (Sect. 3.2), results are reported in terms of FA displacements rather than forces. Note that the calculations for single FA (represented similarly as in the DNM as an ellipse with 2 μm major and 1 μm minor axis) were force-controlled in the sense that a force was imposed to an elliptical area via the definition of a distributed load. Corresponding results in Figs. 10 and 11 refer to the resulting displacement of a point in the center of the ellipse. The dark blue line shows the mean displacement of an FA in the coupled DNM of a single cell. Interestingly, taking the corresponding mean force and applying it to an isolated single FA (black line) leads to less than half the average displacement of the single cell FA, indicating the amplifying effect of the other FAs in a cell. The alternative representation of a cell as a contracting disc (dark dashed line), which agrees with the DNM in terms of area contraction, also leads to maximal displacements at the disc edge that are smaller than the mean FA displacement in their equivalent DNM counterparts. Notably, when performing the same comparison between DNM and contracting disk models for a 13 cell patch (solid and dashed light blue lines) the difference is less.

The normalized values indicate different sensitivities to substrate thickness for the different models (Fig. 10B). The deformation behavior of the single FA is less sensitive to changes in thickness than FAs in the coupled model of a single cell or cell patch, whereas the behavior of contracting discs exhibited larger sensitivity to a change in substrate thickness. A more specific comparison of the induced normalized displacement for different patch sizes on substrates with 5 and 10 μm thickness is presented in Fig. 11. On the substrate with thickness of 5 μm the displacement of a single FA decreased by 5 %, while for coupled models of single cells the reduction was 12 % and for all cell patches 20 %. A disc representative of a single cell exhibits a 23 % decrease and sensitivity increases with disc size, with a disc representative of a cell cluster of 13 cells leading to 48 % decrease in displacement magnitude. Importantly, while the discs exhibit a continuous increase in sensitivity with size, the coupled models do not display size dependence. Thus, using the continuum model, conclusions on thickness sensitivity depend on the disc size, i.e. cell or patch radius.

## Discussion

A DNM of contracting cells and cell patches on continuum flexible elastomer substrates was used to analyze ‘depth sensing’ of contractile cells. The model provides subcellular resolution representing components of the contractile actin cytoskeleton as well as FAs and cell-cell junctions. Parameters to model actin contractility were selected to match observations in two step traction force microscopy experiments (Jakob et al. 2024). The cells’ contractile properties can be conceptualized as that of an actuator with a distinctive force-displacement relationship capable of inducing substrate displacements at the FAs: Maximum force occurs for a rigid substrate, while displacements are large for very compliant elastomers.

### Mechanics at FAs as a cue for cell spreading

The model represents purely mechanical interactions of a cell as an “actuator" on a deformable substrate, which can be investigated on a subcellular scale. It does not account for any mechanobiological processes that may take place in addition. Furthermore, cytoskeletal mechanics of various cell types was observed to change from fluid-like to solid-like depending on the time scale of mechanical loading (Qiao et al. 2025), which is not represented in the present model.

Changes in cell area due to active cellular contraction revealed area changes of few percent, i.e. orders of magnitude smaller than area differences reported in literature for different substrate thicknesses (Tusan et al. 2018; Buxboim et al. 2010; Mullen et al. 2015). This implies that changes in cell area (i.e. cell “spreading") are of active biological nature rather than of purely mechanical one. In fact, it is commonly accepted that cells feel their mechanical surrounding through integrins, which form FAs linking the extracellular space to the cytoskeleton. The FAs have been described as a “molecular clutch" that reinforces its adhesion to stiff substrates and softens its grip on soft ones (Sun et al. 2016). By force-mediated conformational changes of the integrins and its ligands, cryptic binding sites are exposed, leading to FA reinforcement upon an increase in load (Li et al. 2016). This suggests that cell stiffness sensing, and thus changes in cell morphology and spreading upon changes in substrate thickness, are mediated by changes in adhesion forces at specific FA sites. Computational representation of the mechanisms of cell cytoskeletal remodeling leading to cell spreading would require consideration of dynamic formation and repositioning of FAs, which would significantly enhance the complexity of the simulations.

### Isolated FAs, FAs in cells and cell patches

Given this critical role of the FA-length scale, we here studied the sensitivity to substrate thickness in terms of the mean FA displacement and force magnitude. When considering the results for the single cells and different cell patches in terms of the absolute values (Fig. 6A,D), the single cell leads to higher forces and displacements values than the patches. Among the cell patches the force tends to decrease as the patch becomes smaller, but this only weak tendency is even less clear for the displacements. The similarity between the 4 and 8 cell patches indicates that rather than patch size, the randomly created intracellular network structure may govern the magnitude of induced mechanical interactions at FAs. Calculations for a single FA (Fig. 10) led to smaller displacements compared to FAs in cells, and are therefore indicative of the influence of their collective behavior.

Activation of the actin network generally leads to different forces and displacements in the different models for cells and cell patches of different size. One may therefore hypothesize that the observed differences between single cells and patches could result from a non-linear response of the hyperelastic substrate, rather than from a different collective behavior. To test this, we analyzed the response of a single FA upon doubling the applied FA force. As this test revealed a nearly linear relation between force and displacement, we excluded material non-linearity as a main cause for the differences observed in Fig. 6.

To analyze how forces and displacements change relative to those on a substrate of infinite height, we normalized these data by their corresponding values for the 200 μm case. Based on these normalized data, noticeable differences in substrate height sensitivity were only present for FA displacements when comparing the single cell and cell patches, while the force curves remained close, see Fig. 6C,F. Importantly, force increase due to reduced thickness was about 5 % for all cases considered. This may question FA forces as the mechanism of thickness sensing of cells and cell patches. In fact, the change in mean FA force is very small compared to the variability of force magnitudes at FAs, which ranged from several tens of pN to ∼10 nN in the simulations.

The stronger sensitivity on substrate height observed for FA displacements (Fig. 6F) may indicate that cells as actuators are sensitive also to FA displacements, which might be linked to differences in local cytoskeletal deformations associated with different FA movements. With reference to the interpretation of FA as “molecular clutch" (Sun et al. 2016), displacement variations across adjacent FAs is likely to induce larger local strains, affecting the force transmitted through the clutch in each FA, thus leading to a cue detectable via integrins’ activation.

### Substrate strains induced by cells and cell clusters

The computed area strains underneath single cells and cell patches show considerable heterogeneity due to the model’s subcellular resolution on the μm scale. Neighboring cells in a cell patch exhibit considerable interdistance of their FAs. Moreover, FA sites are considerably smaller than the cells themselves. Thus, a separation of scales between FA and single cells or cell patches is expected.

Substrate deformation is induced at FA scale, and cells in clusters induce similar deformation patterns as individual cells on a cell scale. For that reason, a cell patch cannot be considered as just a large actuator in terms of its interaction with the substrate.

When considering a single cell, the observed insensitivity of FA forces or displacements for thicknesses of 5 μm or larger (Fig. 6) correlates with the size of the individual FA, i.e. the order of 2 μm or a multiple thereof.

For cell patches, an outer ring of FAs collectively displayed larger displacements (Fig. 9). We hypothesize that FAs located in the outer ring would “feel" the rigid substrate bottom before FAs in the inner domain, and the length scale of that interaction might be connected to the “radial thickness" of that ring. Importantly, upon an increase of patch size, this characteristic dimension of the ring does not change. This is in line with the observation that depth sensitivity does not change for increasing patch sizes (see e.g. Figs. 5, 6 and 7).

### Continuum vs. subcellular resolution models

When we compared the displacement behavior of the coupled DNM-substrate models of a single cell to the one of a single FA, and to the one of continuous discs, we observed that the sensitivity of the coupled model is closer to the one of the single FA. All these results confirm that substrate thickness-dependent mechanosensing at FA occurs on a smaller than cellular length scale. Consequently, investigations require models with subcellular resolution.

**Fig. 12:**
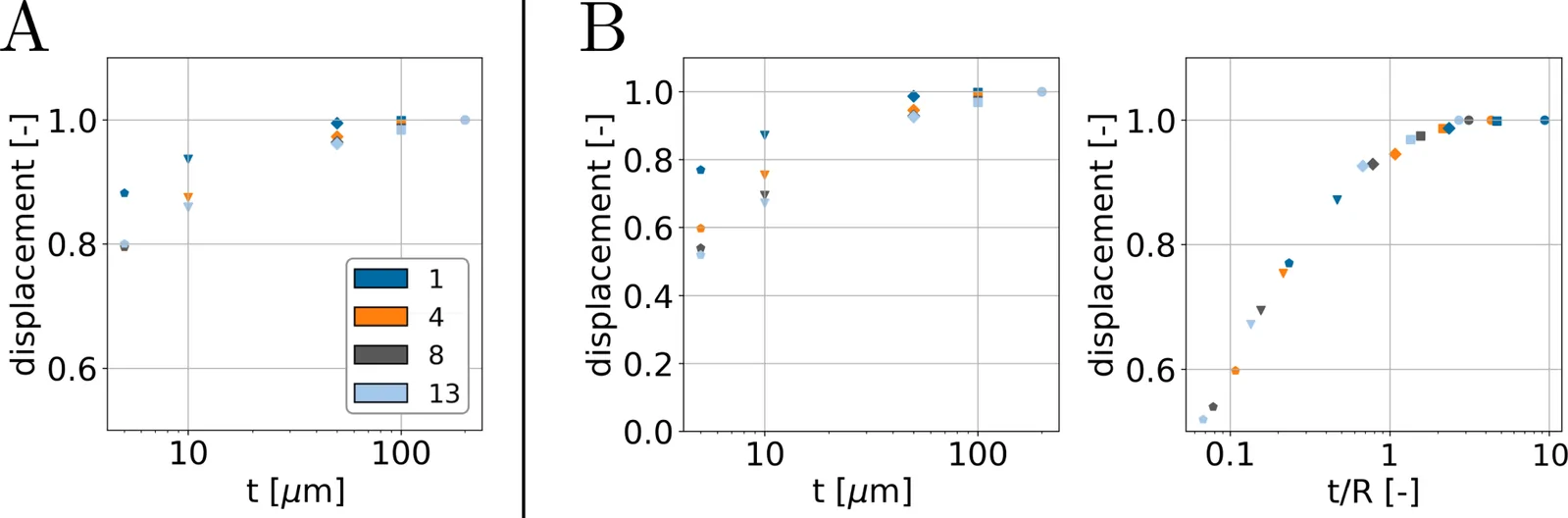
Comparison between the deformation responses to thickness of the hybrid model (**A**) and continuum discs (**B**). **A** Two distinctive behaviors can be observed, one for a single cell (dark blue) and the other for the cell patches with 4 (orange), 8 (dark grey), and 13 (light blue) cells. **B** A clear categorization for continuum representations of cells and cell patches is not possible due to the big scatter (left). However, when normalizing the substrate thickness with the radius of the cell patch, all data falls on one line (right), signifying dependence of the continuum disc deformation response on the cell radius. Circles are displacement values from substrates with 200, squares with 100, diamonds with 50, triangles with 10, and pentagons with 5 μm thickness

When considering the deformation behavior of the disc models on a substrate, dependence on the size of the disc was observed (Fig. 11). To investigate size dependence, we normalized the substrate thickness with the disc radius *R*. As shown in Fig. 12, after normalization, all simulation results fall on one line. As expected, the deformation response of continuum discs depends on their radius. The behavior of the cell and cell patches represented as DNM is therefore fundamentally different to that of an ‘equivalent’ continuum disc.

### Coordinated FA groups as a relevant length scale

As highlighted in the introduction, the main novelty of the present computational analysis is the possibility to represent the highly localized cell-substrate interaction and thus to investigate the length scale governing depth sensing by cells. The corresponding quantifications reported in Fig. 6 indicate that the relevant scale is the one of a group of FAs. This result may be rationalized based on the coordinated concentric contraction observed in (Fig. 8A), which emerges from the tendency of adjacent FAs to move in similar directions. Thus, the force-induced motion of an individual FA is amplified by the substrate deformation induced by neighboring FAs. This interplay might be interpreted as a collective behavior of a “FA cluster", larger in size than the single FA, and yet considerably smaller than the whole cell, see Fig. 9. This mechanism is equally relevant for the formation of the active ring in cell patches, thus leading to a common length scale of thickness sensitivity for individual cells and cell patches.

The present findings are thus in contrast with previous computational studies reporting cell radius or the dimension of cell patches as the relevant scale (Yang et al. 2023; Sen et al. 2009; Lin et al. 2010; Butler et al. 2002; Yang et al. 2006). Maloney et al. (2008) proposed the isolated FA size as relevant scale, which is not confirmed by our calculations. Interestingly our results are in line with Schwarz et al. (2002), which used dimensional analysis to suggest that the relevant dimension is the one of the size of “FAs or a cluster of neighboring FAs if the corresponding forces point in the same direction”.

The results presented in this work are also in line with experimental studies, for which relevant cell adaptation takes place for thickness values in the order of several μm (Tusan et al. 2018; Buxboim et al. 2010; Mullen et al. 2015). Increased sensitivity was suggested for cell patches of several tens to several hundreds of cells in *in vitro* experiments (Tusan et al. 2018), with changes measurable for thicknesses larger than few tens of  μm. Our results also indicate higher depth sensitivity for cell patches but the increase is less pronounced than the one reported in Tusan et al. (2018). Note that the substrate material used in Tusan et al. (2018) was much softer than the one considered in the present study. This influences the displacement magnitudes and correspondingly activates nonlinear geometrical and material effects, thus possibly increasing depth sensitivity, in particular for cell patches that generate larger magnitudes of displacements at their outer edges (cf. Fig. 9).

### Remarks and limitations

The model presented here allows for the characterization of cell-substrate interactions on a subcellular scale. As such, it constitutes an important step towards a better understanding of the variable substrate stiffness perceived by cells due to the substrate’s finite thickness. Despite these merits, several limitations are to be highlighted. First, the stiffness and activation parameters characterizing the mechanical behavior of the coupled models used here were representative for single endothelial cells on flexible elastomers (Lúa 2020). However, for other cell types, different parameters would apply, influencing the behavior of the cell as an actuator. In fact, simulations of other cell types would allow us to assess the influence of their specific cytoskeletal features and contractile behavior on their substrate depth sensing ability. Note also that in the present model the distribution of FAs was considered as fairly uniform (with slightly lower density in the region of the nucleus, in line with Nair et al. (2021)), while recent investigations (Chala et al. 2021) suggest higher numbers of FAs at the cell periphery. A different spatial distribution of FAs could be considered in the present model framework. This would affect simulation results in terms of force and displacement field but it is not expected to influence the general conclusions of this work concerning the length scale of depth sensing. Furthermore, the substrate stiffness of 7 kPa was chosen here based on previous work on traction force microscopy (Lúa 2020). *In vitro* experiments may use considerably softer substrates (∼1 kPa) (e.g. Tusan et al. (2018); Buxboim et al. (2010)) and this may affect depth sensing due to non-linear effects, similarly to changes in DNM properties. Larger local deformations would also increase the dependence of depth sensing on the specific hyperelastic model and its nonlinear strain stiffening behavior, which was not investigated in the present work. Lastly, the models presented here allowed for the representation of purely mechanical effects of cellular contraction on elastomer substrates. It does not account for active biological and biochemical processes. Such processes might be responsible for substantial increase in cell activation or migration, thus influencing thickness-dependent cell-perceived substrate stiffness.

## Conclusions

The substrate stiffness perceived by cells was previously identified as important property influencing cell fate. While lower limits of the cell-perceived stiffness are given by the stiffness of the substrate material, their upper limit is also influenced by the thickness of the substrate within a rigid container. To analyze the dependence of cell-perceived stiffness on substrate height we created a coupled discrete-continuum model that represents the cytoskeletal structures as a discrete network and the substrate as a deformable continuum, whose thickness was varied. Based on the model’s subcellular resolution, cell-substrate interaction could be analyzed on the scale of single FAs. The size of a few FAs was identified as a governing length scale for depth sensing of contracting cells and cell patches. FA force dependence on substrate thickness was weak, whereas sensitivity of FA displacement was larger. This indicates that FA displacement with respect to neighboring FAs or other cytoskeletal components may provide additional sensing mechanisms. Comparisons of the coupled model to a model of a contracting disc revealed fundamentally different responses of the two model types. While the sensitivity of the deformation response in the contracting disc model depended on its radius, coupled models of cell patches with different sizes exhibited negligible differences. The contractile behavior of cell patches modeled with the coupled model caused heterogeneous substrate deformation fields with different pattern for cells and cell patches.

While some of our computational results are in line with previous experiments, such as the critical values of substrate thickness for single cells, other data were only partly reproduced. Future work should consider the influence of substrate material stiffness and non-linearity, as well as differences in cell contractility to better rationalize the observed depth sensitivity of cell clusters. In-vitro studies with endothelial cells cultured on substrates of similar stiffness as the one used for the present analysis should be performed to provide experimental validation of the present computational results.

## Data Availability

Relevant data will be provided by the authors upon reasonable request.
